# First-time postmenopausal bleeding as a clinical marker of long-term cancer risk: A Danish Nationwide Cohort Study

**DOI:** 10.1038/s41416-019-0668-2

**Published:** 2019-12-06

**Authors:** Maria B. Bengtsen, Katalin Veres, Mette Nørgaard

**Affiliations:** Department of Clinical Epidemiology, Institute of Clinical Medicine, Aarhus University Hospital, Olof Palmes Allé 43-45, 8200 Aarhus N, Denmark

**Keywords:** Reproductive signs and symptoms, Epidemiology, Cancer epidemiology

## Abstract

**Background:**

Data on long-term risk of cancer after a postmenopausal bleeding diagnosis are sparse.

**Methods:**

We used Danish medical registries to conduct a population-based cohort study of women with a first hospital-diagnosed postmenopausal bleeding during 1995–2013. We computed the absolute risk of cancer and the standardised incidence ratio (SIR) comparing the observed cancer incidence with that expected in the general population.

**Results:**

Among 43,756 women with postmenopausal bleeding, the absolute 1- and 5-year risk of endometrial cancer were 4.66% and 5.18%, respectively. The SIR of endometrial cancer was elevated during 0–3 months (SIR = 330.36 (95% CI: 315.43–345.81)), 3–12 months (SIR = 11.39 (95% CI: 9.79–13.17)), 1–5 years (SIR = 2.55 (95% CI: 2.19–2.94)) and >5 years of follow-up (SIR = 1.63 (95% CI: 1.40–1.90)). All selected gynaecological and urological, gastrointestinal and haematological cancers had elevated 0–3 months SIRs. Beyond 1 year of follow-up the SIRs of ovarian and bladder cancer remained elevated with a 1–5-year SIR of 2.15 (95% CI: 1.71–2.65) and 1.45 (95% CI: 1.14–1.80), respectively.

**Conclusions:**

In the Danish population, women with a first hospital-diagnosed postmenopausal bleeding have an increased 0–3 months risk of gynaecological, urological, gastrointestinal and haematological cancers. The SIR of endometrial, ovarian and bladder cancer remained elevated for several years.

## Background

Postmenopausal bleeding (PMB) refers to any genital tract bleeding in postmenopausal women, other than that expected during cyclic postmenopausal hormone therapy.^1^ PMB is frequently encountered in both general and hospital settings,^2^ and accounts for ~5% of office gynaecology visits.^3^ The incidence of PMB decreases with increasing time after menopause,^2^ while the risk of an underlying malignancy increases with increasing age.^4^

The most common causes of PMB are benign, such as vaginal atrophy or benign focal lesions.^5^ However, PMB is also the most common presenting sign of endometrial carcinoma. Approximately 5–10% of women with hospital-diagnosed PMB has an underlying endometrial cancer.^4–9^ Although PMB is most commonly attributed to an intrauterine source, it may also originate from other gynaecological or non-gynaecological sources, such as the cervix, vagina, ovaries, bladder, urethra or lower gastrointestinal tract.^10^ Occasionally, PMB also occurs due to non-structural causes such as coagulopathies as seen in haematologic cancers.^11^

While the short-term risk of underlying endometrial cancer has been studied previously,^4–6,8,9^ few studies investigated the long-term risk of gynaecological as well as the risk of non-gynaecological cancers, and these studies were limited by size (<700 participants) and selective inclusion of patients from specific hospitals.^12,13^

We therefore conducted a nationwide population-based cohort study to investigate the long-term risk of cancer and the particular risks of gynaecological, urological, gastrointestinal and haematological cancers after a first-time hospital diagnosis of PMB.

## Methods

### Data sources and study population

We performed a nationwide cohort study in Denmark (5.6 million residents) in the period 1995 through 2013. The Danish National Health Service provides tax-funded medical care to all residents, with free access to treatment at general practitioners, hospitals, outpatient clinics and with reimbursement for prescription drugs.^14^ All Danish residents have a civil registration number that encodes age and gender, and allows unambiguous linkage between medical databases and public registries.^15^

The Danish National Patient Registry (DNPR) captures all admissions to Danish hospitals since 1977, and emergency room and outpatient clinic visits since 1995.^16^ Outpatient visits include visits to hospital-based (ambulatory) specialty clinics, whereas visits to private practice specialists or general practitioners are not included.^16^ In the DNPR, diagnoses are classified according to the International Classification of Diseases (ICD), 8th revision, until the end of 1993, and the 10th revision thereafter.^16^

We used the DNPR to identify a cohort consisting of all women with a primary or secondary inpatient, outpatient or emergency room diagnosis of PMB from 1 January 1995 to 30 November 2013 using the ICD-10 code: N950. We excluded women diagnosed with PMB from 1977 through 1995 (ICD-8 code 626.7) to restrict our cohort to women with a first-time PMB diagnosis.

### Cancer

Cancer incidences were obtained from The Danish Cancer Registry (DCR). This registry has prospectively recorded all cancers diagnosed in Denmark since 1943, classified according to the ICD’s 10th revision, and ICD Oncology codes (ICD-0-1-3) for topography and morphology.^17^ We categorised cancer outcomes as: gynaecological cancers (endometrial cancer, cervical cancer, ovarian cancer, vaginal cancer, and cancer in the external genitalia), urological cancers (bladder cancer and kidney cancer), gastrointestinal cancers (colon cancer, rectal cancer, and liver cancer) and haematological cancers (acute myeloid leukaemia and Non-Hodgkin lymphoma).

We excluded patients diagnosed with cancer (except non-melanoma skin cancer) or endometrial hyperplasia with atypia (since this precancer is treated similar to cancer) prior to their first hospital contact leading to the PMB diagnosis.

### Covariates and confounders

Based on diagnoses recorded in the DNPR prior to the date of the PMB diagnosis, we collected data on the presence of comorbidity according to the Charlson Comorbidity Index (CCI), defined as at least one of the 19 chronic diseases included in the CCI. Furthermore, we retrieved data on previous gynaecological diseases known to cause PMB (including vaginal atrophy, uterine fibroids and polyps, endometrial hyperplasia without atypia, senile vaginitis, and inflammatory diseases of the female genital tract), cardiovascular disease and diabetes mellitus (see Supplementary Table 1 for ICD codes). From the Danish Medical Birth Registry (DMBR), we collected data on nulliparity. The DMBR contains data on all live and stillbirths in Denmark since 1973.^18^

From the Danish National Health Service Prescription Database, we retrieved information on use of hormonal replacement therapy (HRT), defined as at least one reimbursement of a HRT drug (ATC codes: G03C, G03D, G03F and G03CX01) within 6 months before the first PMB diagnosis.

### Statistical analysis

Each woman was followed from the date of the first diagnosis of PMB until the date of the first cancer diagnosis (any type of cancer except non-melanoma skin cancer), death, emigration, or 30 November 2013, whichever came first. We reported the number and proportion of patients in our cohort according to age at time of PMB diagnosis. We calculated median follow-up time and interquartile range (IQR). Next, we estimated the observed/expected (O/E) ratio and standardised incidence ratios (SIR). SIR, a measure of relative risk, compares the observed incidence of cancer among patients with PMB with that expected based on national cancer incidence rates by age (1-year groups) and calendar year (1-year groups). Confidence intervals (CIs) for SIRs were computed assuming a Poisson distribution. We estimated the SIRs for all gynaecological, urological, gastrointestinal and haematological cancers with more than 10 observed cases. We classified the follow-up period as: 0–<3 months, 3 months–<1 year, 1–5 years, and >5 years.

We calculated the absolute risk (the cumulative incidence proportion) of cancer after 3 months, 1 year, and 5 years of follow-up, treating death as a competing risk.^19^

We performed stratified analyses according to age categories (40–49, 50–59, 60–69 and ≥70 years), type of hospital contact (inpatient, outpatient or emergency room), presence of comorbidity according to CCI (yes/no), cardiovascular disease (yes/no), diabetes mellitus (yes/no), PCOS (yes/no), nulliparity (yes/no), previous gynaecological disease (yes/no) and use of HRT (yes/no). For the nulliparity analysis, we restricted the cohort to women born from 1955 and forward, because the DMBR only contains data on births from 1973 and forward.

All statistical analyses were conducted using the SAS statistical software package, v. 9.2 (SAS Institute, Cary, NC).

## Results

We identified 43,756 women with a first-time hospital-based PMB diagnosis. The median age was 59 years (IQR: 54–68). The 43,756 women yielded a total follow-up of 301,927 person-years, corresponding to a median follow-up time of 6 years (IQR: 2–11). In total, we lost 134 women to follow-up (130 emigrated and 4 disappeared). Women were diagnosed during a hospital outpatient clinic visit (82%), inpatient hospital stay (17%), or emergency room visit (1%). The majority (76%) had none of the 19 comorbidities included in the CCI. Among all women, 9,567 (22%) received HRT, 5,023 (11%) had a previous diagnosis of a uterine polyp and 2,965 (7%) fibroma in the uterus, and around 1% had a previous diagnosis of endometrial hyperplasia without atypia, atrophy or inflammatory diseases of the uterus.

### Endometrial cancer

**Fig. 1:**
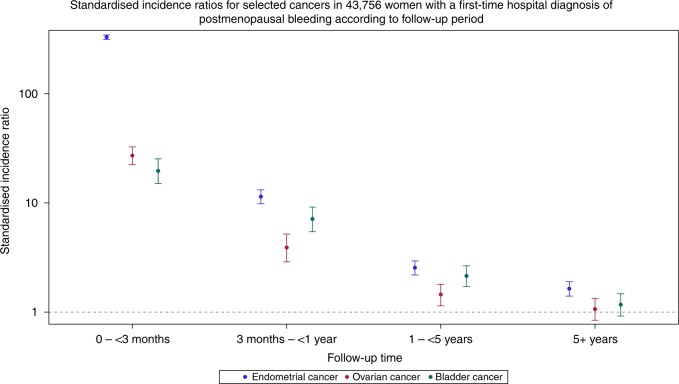
Vertical lines represent 95% confidence intervals.

In total, we observed 2,380 cases of endometrial cancer compared with 201 expected (SIR = 11.86 (95% CI: 11.39–12.35) (Table 1). Within the first 3 months following the PMB diagnosis, 1,839 cases of endometrial cancer were identified compared with 6 expected (SIR = 330.4 (95% CI: 315.43–345.81). The SIR remained elevated from 3 months to 1 year of follow-up (SIR = 11.39 (95% CI: 9.79–13.17) and 1–5 years of follow-up (SIR = 2.55 (95% CI: 2.19–2.94); Fig. 1). After more than 5 years of follow-up, a 60% elevated risk of being diagnosed with endometrial cancer remained compared with women in the general population (SIR = 1.63 (95% CI: 1.40–1.90).

**Table 1:** Number of observed and expected endometrial cancers and standardised incidence ratios with corresponding 95% confidence intervals in 43,756 women with a first-time hospital diagnosis of postmenopausal bleeding, stratified by follow-up period and patient characteristics.

Patient characteristics	No. of patients (%)	Follow-up period							
		<3 months		3 months – <1 year		1–5 years		>5 years	
		Cancers O/E	SIR (95% CI)	Cancers O/E	SIR (95% CI)	Cancers O/E	SIR (95% CI)	Cancers O/E	SIR (95% CI)
All	43756 (100)	1839/6	330.36 (315.43–345.81)	182/16	11.39 (9.79–13.17)	185/73	2.55 (2.19–2.94)	174/106	1.63 (1.40–1.90)
Age									
40–49	3095 (7)	7/0	81.23 (32.57–167.33)	N/A	N/A	N/A	N/A	11/7	1.61 (0.80–2.88)
50–59	19840 (45)	302/2	161.85 (144.11–181.18)	30/6	5.32 (3.59–7.59)	52/30	1.75 (1.31–2.29)	84/56	1.51 (1.20–1.87)
60–69	12004 (27)	612/2	306.79 (282.97–332.09)	64/6	11.12 (8.57–14.21)	73/25	2.87 (2.25–3.61)	49/32	1.51 (1.12–2.00)
≥70	8817 (20)	918/2	566.72 (530.65–604.60)	87/4	20.24 (16.21–24.97)	56/15	3.63 (2.74–4.72)	30/12	2.60 (1.75–3.71)
Type of hospital contact									
Inpatient	7505 (17)	370/1	365.65 (329.34–404.88)	31/3	10.65 (7.24–15.12)	26/13	1.94 (1.27–2.85)	41/24	1.70 (1.22–2.31)
Outpatient	35969 (82)	1446/5	319.56 (303.30–336.46)	147/13	11.31 (9.56–13.30)	157/59	2.67 (2.27–3.12)	131/82	1.60 (1.34–1.90)
Emergency room	282 (1)	23/0	771.58 (488.96–1157.8)	N/A	N/A	N/A	N/A	N/A	N/A
Medical history									
Charlson Comorbidity Index									
CCI = 0	33257 (76)	1345/4	331.47 (313.99–349.67)	127/12	10.76 (8.97–12.80)	135/56	2.40 (2.01–2.84)	151/90	1.68 (1.42–1.97)
CCI ≥ 1	10499 (24)	494/2	327.36 (299.12–357.54)	55/4	13.18 (9.93–17.15)	50/16	3.05 (2.26–4.02)	23/17	1.39 (0.88–2.09)
Cardiovascular disease									
Yes	11034 (25)	624/2	383.64 (354.12–414.96)	60/5	13.30 (10.15–17.12)	59/17	3.47 (2.64–4.48)	31/14	2.17 (1.47–3.08)
No	32722 (75)	1215/4	308.36 (291.26–326.20)	122/11	10.64 (8.83–12.70)	126/56	2.26 (1.89–2.70)	143/92	1.55 (1.31–1.83)
Diabetes mellitus									
Yes	2433 (6)	191/0	557.96 (481.63–642.95)	16/1	17.30 (9.88–28.10)	21/3	6.34 (3.92–9.69)	9/3	3.37 (1.54–6.39)
No	41323 (94)	1648/5	315.44 (300.39–331.05)	166/15	11.03 (9.41–12.84)	164/69	2.37 (2.02–2.76)	165/104	1.59 (1.36–1.85)
Previous gynaecological disease^a^									
Yes	8207 (19)	237/1	222.92 (195.44–253.18)	36/3	11.66 (8.16–16.14)	33/13	2.48 (1.70–3.48)	25/15	1.64 (1.06–2.41)
No	35549 (81)	1602/5	355.72 (338.51–373.58)	146/13	11.33 (9.56–13.32)	152/59	2.56 (2.17–3.00)	149/91	1.63 (1.38–1.92)
Nulliparity^b^									
Yes	5662 (85)	19/0	319.31 (192.15–498.67)	N/A	N/A	N/A	N/A	N/A	N/A
No	1036 (15)	29/0	83.22 (55.72–119.52)	N/A	N/A	N/A	N/A	N/A	N/A
Use of hormonal replacement therapy^c^									
Yes	10091 (23)	283/1	196.15 (173.96–220.39)	37/4	8.93 (6.28–12.30)	29/18	1.61 (1.08–2.31)	37/18	2.06 (1.45–2.84)
No	33665 (77)	1556/4	377.31 (358.79–396.53)	145/12	12.25 (10.34–14.42)	156/55	2.86 (2.43–3.34)	137/89	1.55 (1.30–1.83)

Cancer occurring with less than five observed cases were included in the main analysis, but not analysed separately (i.e. reported as N/A)

*O/E* observed/expected, *SIR* standardised incidence ratio, *CI* confidence interva, *CCI* Charlson Comorbidity Index

^a^Previous gynaecological disease known to cause postmenopausal bleeding

^b^The nulliparity analysis was restricted to patients born after 1955 because the Danish Medical Birth Register only contains data on births from 1973 and forward

^c^Use of hormonal replacement therapy was defined as reimbursement of at least one hormonal replacement therapy drug within 6 months before postmenopausal bleeding diagnosis

The absolute risk of endometrial cancer was 4.23% at 3 months, 4.66% at 1 year and 5.18% at 5 years follow-up after the PMB diagnosis when considering death as a competing risk (Table 2).

**Table 2:** Absolute risk of endometrial cancer at 3 months, 1 year and 5 years of follow-up in 43,756 women with a first-time hospital diagnosis of postmenopausal bleeding, stratified by patient characteristics.

Patient characteristics	Follow-up period		
	3 months % (95% CI)	1 year % (95% CI)	5 years % (95% CI)
Overall	4.23 (4.04–4.42)	4.66 (4.46–4.86)	5.18 (4.97–5.39)
Age			
40–49	0.23 (0.10–0.46)	0.26 (0.13–0.50)	0.41 (0.23–0.70)
50–59	1.53 (1.37–1.71)	1.69 (1.51–1.87)	2.01 (1.82–2.22)
60–69	5.12 (4.74–5.53)	5.68 (5.27–6.10)	6.44 (6.00–6.90)
≥70	10.47 (9.84–11.12)	11.50 (10.84–12.18)	12.26 (11.58–12.96)
Type of hospital contact			
Inpatient	4.94 (4.47–5.45)	5.36 (4.87–5.89)	5.75 (5.24–6.30)
Outpatient	4.05 (3.85–4.25)	4.47 (4.26–4.69)	5.02 (4.79–5.25)
Emergency room	8.18 (5.35–11.75)	9.61 (6.52–13.40)	10.34 (7.12–14.23)
Comorbidity			
Charlson comorbidity index			
CCI = 0	4.07 (3.86–4.28)	4.46 (4.24–4.69)	4.96 (4.72–5.20)
CCI ≥ 1	4.74 (4.34–5.15)	5.28 (4.86–5.73)	5.88 (5.43–6.35)
Cardiovascular disease			
Yes	5.70 (5.27–6.14)	6.27 (5.83–6.74)	6.97 (6.50–7.47)
No	3.73 (3.53–3.94)	4.12 (3.90–4.34)	4.58 (4.35–4.81)
Diabetes mellitus			
Yes	7.89 (6.86–9.01)	8.59 (7.51–9.75)	9.75 (8.58–11.01)
No	4.01 (3.82–4.20)	4.43 (4.23–4.63)	4.91 (4.70–5.13)
Previous gynaecological disease^a^			
Yes	2.91 (2.56–3.29)	3.37 (2.99–3.78)	3.91 (3.49–4.36)
No	4.53 (4.32–4.75)	4.95 (4.73–5.18)	5.47 (5.23–5.71)
Use of hormonal replacement therapy^b^			
Yes	2.82 (2.51–3.16)	3.20 (2.87–3.56)	3.59 (3.23–3.97)
No	4.65 (4.43–4.88)	5.09 (4.86–5.33)	5.65 (5.41–5.91)

*CI* confidence interval, *CCI* Charlson Comorbidity Index

^a^Previous gynaecological disease known to cause postmenopausal bleeding

^b^Use of hormonal replacement therapy was defined as reimbursement of at least one hormonal replacement therapy drug within 6 months before postmenopausal bleeding diagnosis

Increasing age was associated with increasing absolute and relative risk of endometrial cancer. While the absolute 1- and 5-year risks of endometrial cancer for women aged 40–49 years were 0.26% and 0.41%, the corresponding absolute risks for patients aged ≥70 were 11.50% and 12.26%, respectively.

Among patients with a previous diagnosis of cardiovascular disease or diabetes mellitus, PMB was associated with a 2–3-fold elevated risk of endometrial cancer after more than 5 years of follow-up (Table 1). Users of HRT had lower SIRs and absolute risks of endometrial cancer, compared with non-users.

### Cervical cancer, ovarian cancer, vaginal cancer and cancer in the external genitalia

Within 3 months of follow-up after PMB, we observed 203 cases of cervical cancer (SIR = 110.52 (95% CI: 94.84-126.81)), 117 cases of ovarian cancer (SIR = 27.17 (95% CI: 22.47–32.56)), 10 cases of vaginal cancer (SIR = 72.13 (95% CI: 34.53–132.66)) and 8 cases of cancer in the external genitalia (SIR = 14.95 (95% CI: 6.44–29.45)) (Table 3). The SIR of ovarian cancer remained elevated during 3–12 months (SIR = 3.91 (95% CI: 2.88–5.18) and 1–5 years of follow-up (SIR = 1.45 (95% CI: 1.14–1.80). Hereafter, it decreased to that expected compared with the general population. Risks of cervical and vaginal cancer remained elevated during 3 months to 1 year of follow-up. At 5 years of follow-up, the absolute risk of cervical cancer was 0.56% and 0.61% of ovarian cancer (Table 4).

**Table 3:** Number of observed and expected cancers and standardised cancer incidence with corresponding 95% confidence intervals in 43,756 women with a first-time hospital diagnosis of postmenopausal bleeding, stratified by follow-up period and cancer type.

Cancer type	Follow-up period							
	<3 months		3 months –<1 year		1–5 years		>5 years	
	O/E	SIR (95% CI)	O/E	SIR (95% CI)	O/E	SIR (95% CI)	O/E	SIR (95% CI)
Gynaecological cancers	2177/12	175.79 (168.48–183.33)	250/35	7.08 (6.23–8.01)	296/156	1.90 (1.69–2.13)	274/216	1.27 (1.12–1.43)
Endometrial cancer	1839/6	330.36 (315.43–345.81)	182/16	11.39 (9.79–13.17)	185/73	2.55 (2.19–2.94)	174/106	1.63 (1.40–1.90)
Cervical cancer	203/2	110.52 (95.84–126.81)	15/5	2.91 (1.63–4.81)	22/21	1.06 (0.66–1.60)	10/24	0.42 (0.20–0.77)
Ovarian cancer	117/4	27.17 (22.47–32.56)	48/12	3.91 (2.88–5.18)	78/54	1.45 (1.14–1.80)	77/72	1.06 (0.84–1.33)
Vaginal cancer	10/0	72.13 (34.53–132.66)	N/A	N/A	N/A	N/A	N/A	N/A
Cancer in the external genitalia	8/1	14.95 (6.44–29.45)	N/A	N/A	7/7	1.03 (0.41–2.13)	10/11	0.94 (0.45–1.73)
Urological cancers	67/5	14.81 (11.48–18.81)	67/13	5.17 (4.01–6.56)	101/60	1.69 (1.38–2.06)	106/94	1.13 (0.92–1.36)
Bladder cancer	59/3	19.70 (14.99–25.41)	61/9	7.11 (5.44–9.13)	85/40	2.15 (1.71–2.65)	74/63	1.17 (0.92–1.47)
Kidney cancer	8/2	5.23 (2.26–10.31)	6/4	1.37 (0.50–2.99)	16/20	0.80 (0.46–1.30)	32/31	1.04 (0.71–1.46)
Gastrointestinal cancers	58/13	4.35 (3.30–5.62)	66/38	1.73 (1.34–2.21)	184/174	1.06 (0.91–1.22)	271/283	0.96 (0.85–1.08)
Colon cancer	45/9	5.04 (3.68–6.75)	43/25	1.69 (1.23–2.28)	124/117	1.06 (0.88–1.27)	188/193	0.98 (0.84–1.13)
Rectal cancer	13/4	3.46 (1.84–5.92)	20/11	1.86 (1.13–2.87)	49/49	1.00 (0.74–1.32)	69/77	0.90 (0.70–1.13)
Liver cancer	N/A	N/A	N/A	N/A	11/9	1.29 (0.64–2.31)	14/13	1.04 (0.57–1.75)
Haematological cancers	13/5	2.77 (1.47–4.74)	15/13	1.12 (0.63–1.84)	60/61	0.98 (0.75–1.26)	100/97	1.03 (0.84–1.25)
AML	N/A	N/A	N/A	N/A	17/10	1.75 (1.02–2.80)	18/15	1.23 (0.73–1.94)
Non-Hodgkin lymphoma	11/4	2.81 (1.40–5.02)	11/11	0.98 (0.49–1.75)	43/52	0.83 (0.60–1.12)	82/83	0.99 (0.79–1.23)

Cancer occurring with less than five observed cases were included in the main analysis, but not analysed separately (i.e. reported as N/A)

O/E observed/expected, SIR standardised incidence ratio, CI confidence interval

**Table 4:** Absolute risk (cumulative incidence) of gynaecological, urological, gastrointestinal and haematological cancers at 3 months, 1 year and 5 years in 43,756 women with a first-time hospital diagnosis of postmenopausal bleeding, stratified by cancer type.

Cancer type	Follow-up period		
	3 months % (95% CI)	1 year % (95% CI)	5 years % (95% CI)
Gynaecological cancers	5.02 (4.82–5.23)	5.62 (5.41–5.84)	6.49 (6.25–6.72)
Endometrial cancer	4.23 (4.04–4.42)	4.66 (4.46–4.86)	5.18 (4.97–5.39)
Cervical cancer	0.47 (0.41–0.53)	0.50 (0.44–0.57)	0.56 (0.49–0.64)
Ovarian cancer	0.27 (0.22–0.32)	0.38 (0.33–0.45)	0.61 (0.53–0.69)
Vaginal cancer	0.02 (0.01–0.04)	0.03 (0.02–0.05)	0.04 (0.03–0.06)
Cancer in the external genitalia	0.02 (0.01–0.04)	0.02 (0.01–0.04)	0.04 (0.03–0.07)
Urological cancers	0.16 (0.12–0.20)	0.32 (0.27–0.37)	0.61 (0.54–0.69)
Bladder cancer	0.14 (0.10–0.17)	0.28 (0.23–0.34)	0.52 (0.45–0.60)
Kidney cancer	0.02 (0.01–0.04)	0.03 (0.02–0.05)	0.08 (0.05–0.11)
Gastrointestinal cancers	0.13 (0.10–0.17)	0.30 (0.25–0.35)	0.85 (0.76–0.94)
Colon cancer	0.10 (0.08–0.14)	0.21 (0.17–0.25)	0.56 (0.49–0.64)
Rectal cancer	0.03 (0.02–0.05)	0.08 (0.06–0.11)	0.22 (0.18–0.28)
Liver cancer	0.00 (.-.)	0.01 (0.00–0.02)	0.04 (0.02–0.06)
Haematological cancers	0.03 (0.02–0.05)	0.07 (0.05–0.10)	0.25 (0.20–0.30)
AML	0.00 (0.00–0.02)	0.01 (0.01–0.03)	0.06 (0.04–0.09)
Non-Hodgkin lymphoma	0.03 (0.01–0.04)	0.05 (0.03–0.08)	0.18 (0.14–0.23)

*CI* confidence interval

### Urological cancers

Within 0–3 months of follow-up, we found an elevated risk of bladder cancer (SIR = 19.70 (95% CI: 14.99–25.41) and kidney cancer (SIR = 5.23 (95% CI: 2.26–10.31). The risk of bladder cancer remained elevated during 3 months to 1 year (SIR = 7.11 (95% CI: 5.44–9.13) and 1 to 5 years (SIR = 2.15 (95% CI: 1.71–2.65) of follow-up. The absolute risk of bladder cancer was 0.28% after 1 year and 0.52% after 5 years of follow-up.

### Gastrointestinal cancers

The 3-month SIRs of colon and rectal cancer following a first-time episode of PMB were 5.04 (95% CI: 3.68–6.75) and 3.46 (95% CI: 1.84–5.92), respectively. The SIR remained slightly elevated during 3 months to 1 year of follow-up, and then decreased to that expected in the general population.

### Haematological cancers

The risk of haematological cancers following an episode of PMB was elevated during 0–3 months (SIR = 2.77 (95% CI: 1.47–4.74)), and hereafter it did not differ from that expected in the general population.

## Discussion

In this Danish population-based study, we found that a first-time hospital-diagnosed PMB is a clinical marker of gynaecological, urological, gastrointestinal and haematological cancer. The risk of cancer was particularly high within the first three months of follow-up but remained elevated for several years after diagnosis for endometrial, bladder and ovarian cancer.

Urogenital cancers, gastrointestinal cancers and haematologic cancers could all be associated with PMB through direct tumour invasion of the female genital tract or through tumour-induced coagulopathy. Moreover, an association could occur if bleeding from non-gynaecological tumours are misinterpreted as genital tract bleeding. This is, to our knowledge, the first nationwide cohort study estimating the risk of cancers other than endometrial cancer following a first-time episode of PMB, and the first study to evaluate the risk of cancer according to follow-up time. Visser et al.^13^ investigated the risk of endometrial cancer after PMB among 668 women with PMB from January 2009 to April 2011 at two different hospitals in the Netherlands. Initially, 73 (10.9%) were diagnosed with endometrial cancer. During a follow-up period of more than 3 years, an additional 8 women were diagnosed with endometrial cancer. Similarly, Gull et al.^12^ investigated the risk of cancer among 339 women with PMB referred to a single hospital in Sweden from November 1987 to October 1990. Initial examination lead to diagnosis of endometrial cancer in 39 women (11.5%) and during a 10-year follow-up period, 7 women were additionally diagnosed with endometrial cancer. Our study extends these findings of the cancer risk after PMB diagnosis, with a quantification of the absolute and relative long-term risk of endometrial cancer as well as other gynaecological and non-gynaecological cancers following a first-time PMB.

Our finding of an elevated risk of all the investigated cancers (except acute myeloid leukaemia and liver cancer) within the first 3 months of follow-up after a first-time PMB diagnosis most likely reflects PMB being a presenting symptom of occult cancer or occult cancer diagnosed during work-up for PMB. However, the risk of endometrial cancer, ovarian cancer and bladder cancer remained elevated for more than 1 year after the PMB diagnosis, suggesting that heightened diagnostic effort is not the entire explanation. The persistently increased SIR beyond 1 year of follow-up might represent “missed” cancers that could have been detected during work-up for PMB. As PMB is the most common presenting sign of endometrial cancer, women diagnosed with endometrial cancer during follow-up will often present with recurrent bleeding episodes before their diagnosis of endometrial cancer.^12,13^ However, the persistently elevated SIR of ovarian and bladder cancer requires attention and suggest the need for a more a broadened diagnostic work-up for cancer in patients with normal gynaecological findings after initial work-up.

In our study, the absolute 5-year risk of endometrial cancer was 5.2%, which is relatively low compared with the previously often reported 10% incidence of endometrial cancer after initial work-up for PMB.^6,7,9,12,13^ The Danish coding practice could be a part of the explanation, since women with an obvious underlying cause of PMB at first hospital contact, might only receive the code of the underlying cause without a PMB diagnosis code. For this reason, the present PMB population is likely to represent women with no obvious cause of PMB after initial examination. Moreover, we excluded patients with prior cancer diagnosis because they have an elevated risk of secondary cancers overall.^20^ Exclusion of women with prior cancer also leads to exclusion of a group of women who are predisposed to endometrial cancer, including breast cancer patients treated with Tamoxifen and patients with colorectal cancer related to HNPCC. Consequently, this exclusion is also likely to lead to a lower cancer incidence compared with studies that did not exclude these patients.^4–9,12,13^

Our study has several strengths. Access to Danish nationwide medical registries allowed us to conduct a large population-based study with virtually complete follow-up. Due to the high completeness of incident cancers in the DCR,^17^ we believe to have detected virtually all cases of cancers in our cohort. Still, some weaknesses need to be addressed. We identified our cohort based on diagnoses registered in the DNPR, and these might not be entirely accurate. Still, as the positive predictive value of coding lower gastrointestinal bleeding has been reported to 96% in a study that included both in- and outpatients and was based on DNPR data retrieved in the period 2004–2011,^21^ we do not think that misclassification of PMB constitutes a major source of bias in our study. Due to lacking information on endometrial thickness and body mass index, we could not examine whether the risk of cancer in women with PMB varied according to these variables.

The Danish Society of Obstetrics and Gynecology provides national guidelines to ensure uniform work-up of women with PMB across the country. Since 2008, women presenting with postmenopausal bleeding should be referred for further investigation through a Danish national integrated cancer pathway.^22^ This referral triggers prompt investigation by a specialist in gynaecology and should, according to guidelines, include gynaecological examination, abdominal palpation and vaginal ultrasound.^23^ In cases with endometrial thickness in >4 mm or irregular endometrium endometrial sampling is recommended. Otherwise, no further investigation of the endometrium is recommended, unless rebleeding occurs.^22^ Despite the existence of national integrated pathways, we cannot be sure that all women were referred for or underwent appropriate diagnostic work-up according to national guidelines, which is a study weakness.

We used SIRs as a measure of relative risk, comparing the risk of cancer in our PMB cohort to that expected in the general population. Unlike some women in the general population, women in our cohort are unlikely to have undergone hysterectomy upon the time of PMB diagnosis, which could potentially lead to overestimation of the risk of cancer. Correction for hysterectomy was performed in a Danish study investigating the incidence of cervical cancer.^24^ The overall incidence of cervical changed from 17.8/100,000 person-years to 19.3/100,000 person-years after correction for hysterectomies, corresponding to an overall increase of 8.4% in the incidence rate after correction for hysterectomies. The magnitude of this change is not able to explain our SIRs of endometrial cancer of 2.55 during 1–5 years of follow-up and 1.63 after >5 years of follow-up. Moreover, women in our cohort can undergo hysterectomy during follow-up, diminishing the impact of correction for hysterectomy.

In conclusion, a hospital-diagnosed PMB is a marker of a long-term risk of urogenital cancer in the Danish population. The sustained elevated SIR of ovarian and bladder cancer for several years after PMB diagnosis, suggest a need to broaden the diagnostic work-up in terms of normal gynaecological findings.

## Supplementary information

Supplementary table 1

## Data Availability

Data are available as presented in the paper. According to Danish legislation, our own approvals to use the Danish data sources for the current study do not allow us to distribute or make patient data directly available to other parties. Interested researchers may apply for data access through the Research Service at the Danish Health Data Authority. Up-to-date information on data access is available online (http://sundhedsdatastyrelsen.dk/da/forskerservice). Access to data from the Danish Health Data Authority requires approval from the Danish Data Protection Agency (https://www.datatilsynet.dk/english/the-danish-data-protection-agency/introduction-to-the-danish-data-protection-agency/). We do not have special access privileges to these data.
