# Supplementary material for: First-time postmenopausal bleeding as a clinical marker of long-term cancer risk: A Danish Nationwide Cohort Study
Source: Br J Cancer. 2019 Dec 6;122(3):445–51. doi: 10.1038/s41416-019-0668-2 (PMC7000815; doi:10.1038/s41416-019-0668-2)
Supplement: Supplementary file 1 — Supplementary table 1 [file 41416_2019_668_MOESM1_ESM.docx]

| **Supplementary table 1** ICD-codes defining Charlson Comorbidity Index (CCI) diseases, cardiovascular diseases, and previous gynecological diseases | | |
| --- | --- | --- |
|  | **ICD-8 codes** | **ICD-10 codes** |
| **CCI disease category** |  |  |
| Myocardial infarction | 410 | I21,I22,I23 |
| Congestive heart failure | 427.09, 427.10, 427.11, 427.19, 428.99, 782.49 | I50, I11.0, I13.0, I13.2 |
| Peripheral vascular disease | 440, 441, 442, 443, 444, 445 | I70, I71, I72, I73, I74, I77 |
| Cerebrovascular disease | 430-438 | I60-I69, G45, G46 |
| Dementia | 290.09-290.19, 293.09 | F00-F03, F05.1, G30 |
| Chronic pulmonary disease | 490-493, 515-518 | J40-J47, J60-J67, J68.4, J70.1, J70.3, J84.1, J92.0, J96.1, J98.2, J98.3 |
| Connective tissue disease | 712, 716, 734, 446, 135.99 | M05, M06, M08, M09,M30,M31, M32, M33, M34, M35, M36, D86 |
| Ulcer disease | 530.91, 530.98, 531-534 | K22.1, K25-K28 |
| Mild liver disease | 571, 573.01, 573.04 | B18, K70.0-K70.3, K70.9, K71, K73, K74, K76.0 |
| Diabetes mellitus | 249.00, 249.06, 249.07, 249.09, 250.00, 250.06, 250.07, 250.09 | E10.0, E10.1, E10.9, E11.0, E11.1, E11.9 |
| Hemiplegia | 344 | G81, G82 |
| Moderate to severe renal disease | 403, 404, 580-583, 584, 590.09, 593.19, 753.10-753.19, 792 | I12, I13, N00-N05, N07, N11, N14, N17-N19, Q61 |
| Diabetes with end organ damage | 249.01-249.05, 249.08, 250.01-250.05, 250.08 | E10.2-E10.8, E11.2-E11.8 |
| Any tumor | 140-194 | C00-C75 |
| Leukemia | 204-207 | C91-C95 |
| Lymphoma | 200-203, 275.59 | C81-C85, C88, C90, C96 |
| Moderate to severe liver disease | 070.00, 070.02, 070.04, 070.06, 070.08, 573.00, 456.00-456.09 | B15.0, B16.0, B16.2, B19.0, K70.4, K72, K76.6, I85 |
| Metastatic solid tumor | 195-198, 199 | C76-C80 |
| AIDS | 079.83 | B21-B24 |
| **Cardiovascular diseases** |  |  |
| Acute myocardial infarction | 410 | I21 |
| Stent thrombosis | N/A | T823D, T823E |
| Angina pectoris | 411, 413 | I20, I251, I259 |
| Heart failure | 42709, 42710, 42711, 42719, 42899, 78249 | I500, I501, I502, I503, I508, I509, I110, I130, I132, I420, I426, I427, I428, I429 |
| Hypertension | 400-404 | I10-I15 |
| Cardiac arrhythmias | 42720, 42721, 42722, 42723, 42793, 42794 , 42797, 42791 | I440, I441, I442, I443, I455A, I455B, I455C, I455G, I470, I472, I48, I490 |
| Heart valve diseases | 394, 395 | I05, I06, I34, I35, I390, I391, I511A |
| Cardiac inflammation and infection | 39109, 393, 420-423 | I090, I30-I33, I38, I398, I40, I41, I514 |
| Hypercholesterolemia | 27200 | E780 |
| Aorta diseases | 44109-44111, 44119-44121, 44129, 44199 | I710-I716, I718-I719 |
| Intermittent claudication | 44389-44399 | I739A |
| Stroke | 430-431, 433-435 | 160-161, 163-164, G459 |
| **Previous gynecological disease known to cause PMB** | | |
| Atrophy (vaginal or endometrial) | 622.1, 625.09, 629.92, 629.93 | DN858C, DN898B, DN952 |
| Endometrial hyperplasia without atypia | 625.20 | DN850 |
| Polyps in the female genital organs | 625.21 | DN84 |
| Uterine fibroids | 218.99 | DD25 |
| Inflammatory diseases of the uterus | 622 | DN71 |
| Abbreviations: AIDS - Acquired Immune Deficiency Syndrome, ICD - International Classification of Diseases, N/A - Not Available | | |
|  |  |  |
